# Antinociceptive Behavior, Glutamine/Glutamate, and Neopterin in Early-Stage Streptozotocin-Induced Diabetic Neuropathy in Liraglutide-Treated Mice under a Standard or Enriched Environment

**DOI:** 10.3390/ijms251910786

**Published:** 2024-10-08

**Authors:** Pavlina Gateva, Milen Hristov, Natasha Ivanova, Debora Vasileva, Alexandrina Ivanova, Zafer Sabit, Todor Bogdanov, Sonia Apostolova, Rumiana Tzoneva

**Affiliations:** 1 Department of Pharmacology and Toxicology, Faculty of Medicine, Medical University of Sofia, 1431 Sofia, Bulgaria; mhristov@medfac.mu-sofia.bg (M.H.); natasha_nm@yahoo.com (N.I.); debi.vassileva@gmail.com (D.V.); 101975@students.mu-sofia.bg (A.I.); 2 Institute of Neurobiology, Bulgarian Academy of Sciences, 1113 Sofia, Bulgaria; 3 Department of Pathophysiology, Faculty of Medicine, Medical University of Sofia, 1431 Sofia, Bulgaria; zsabit@medfac.mu-sofia.bg; 4 Department of Medical Physics and Biophysics, Medical University of Sofia, 1431 Sofia, Bulgaria; tbogdanov@medfac.mu-sofia.bg; 5 Institute of Biophysics and Biomedical Engineering, Bulgarian Academy of Sciences, 1113 Sofia, Bulgaria; sonia_apostolova@yahoo.com (S.A.); tzoneva@bio21.bas.bg (R.T.)

**Keywords:** liraglutide, neopterin, glutamine-to-glutamate ratio, streptozotocin, neuropathy, diabetes, enriched environment, mice

## Abstract

Diabetic neuropathy (DN) is a common complication of long-lasting type 1 and type 2 diabetes, with no curative treatment available. Here, we tested the effect of the incretin mimetic liraglutide in DN in mice with early-stage type 1 diabetes bred in a standard laboratory or enriched environment. With a single i.p. injection of streptozotocin 150 mg/kg, we induced murine diabetes. Liraglutide (0.4 mg/kg once daily, i.p. for ten days since the eighth post-streptozotocin day) failed to decrease the glycemia in the diabetic mice; however, it alleviated their antinociceptive behavior, as tested with formalin. The second phase of the formalin test had significantly lower results in liraglutide-treated mice reared in the enriched environment vs. liraglutide-treated mice under standard conditions [2.00 (0.00–11.00) vs. 29.00 (2.25–41.50) s, *p* = 0.016]. Liraglutide treatment, however, decreased the threshold of reactivity in the von Fray test. A significantly higher neopterin level was demonstrated in the diabetic control group compared to treatment-naïve controls and the liraglutide-treated diabetic mice (*p* < 0.001). The glutamine/glutamate ratio in both liraglutide-treated groups, either reared under standard conditions (*p* = 0.003) or an enriched environment (*p* = 0.002), was significantly higher than in the diabetic controls. This study demonstrates an early liraglutide effect on pain sensation in two streptozotocin-induced diabetes mouse models by reducing some inflammatory and oxidative stress parameters.

## 1. Introduction

Peripheral neuropathy is one of the most common complications of diabetes, with a prevalence of up to 50–60% [1]. Both type 1 and type 2 diabetes can lead to the development of diabetic neuropathy (DN). Diabetic peripheral neuropathy shows features of progressive, distal-to-proximal degeneration of peripheral nerves, leading to pain and weakness followed by the loss of sensation [2].

Several triggering factors for DN have already been revealed, i.e., hyperglycemia, concomitant dyslipidemia, impaired insulin signal transduction [3], and even hypoglycemia [4]. Being a hallmark of microangiopathic damage, DN is associated with the dysregulation of several pathways, e.g., advanced glycation end-products and their receptors (AGE/RAGE), altered transcriptional factors, pathological collagen crosslinks, reactive oxygen species (ROS) production, and inflammation. ROS are overproduced by AGE/RAGE, the polyol pathway, protein kinase C, and the hexosamine pathway [5]. Low-grade inflammation is triggered by Toll-like receptors (TLR)-2 and TLR-4-cascades, increasing cytokine release and exacerbating fatty acid-induced insulin resistance [6]. Impaired vascular repair/regeneration is indicated by a decrease in the endothelial colony-forming cells [7] and endothelial dysfunction via the dysregulation of poly(ADP-ribose) polymerase (PARP), nuclear factor kappa B (NF-kB), and by Notch-1 pathways [8] accelerating the microvascular damage of neuronal vessels [9].

The incretin system comprises glucagon-like peptide 1 (GLP-1) and gastric-inhibiting peptide (GIP), intestinal hormones released in response to food intake by the mouth. Incretins (GLP-1 and GIP) regulate insulin release, inhibit glucagon, suppress appetite, and decrease stomach motility. Since the establishment of incretin-based therapy more than 15 years ago, the pharmacological studies of incretin mimetics have expanded far beyond their first approved indication as antidiabetic and appetite suppressors [10]. Many of the above-mentioned pathogenic mechanisms of DN are targeted by incretin mimetics, i.e., they reduce ROS generation and AGE receptors [11], mitigate the effect of hyperglycemia on endothelial cells, reduce endothelial inflammation [12], decrease autophagy, and improve endothelial function [13].

The long-acting incretin mimetic liraglutide, an agonist of glucagon-like peptide-1 receptors, is extensively studied not only as a treatment option for type 2 diabetes, obesity, and related complications [14], but also for type 1 diabetes [15]. Data about liraglutide’s ability to improve DN in rats are convincing [16]; however, this has failed to have been reproduced in humans with DN, despite some anti-inflammatory effects [17]. The timing of liraglutide intervention is likely essential, and an early start of liraglutide treatment would be better suited for the neuropathy.

Some nonpharmacological factors referred to as an “enriched environment” (EE) have a beneficial effect on the autonomic nervous system in mice [18]. The protective role of the EE for developing early diabetic optic neuritis has been demonstrated in rodents [19]. An EE refers to specific living conditions with complex, non-stressful, challenging environments that stimulate mental and physical health. An EE is achieved by providing more living space, physical activity, and social relationships, resulting in increased sensory, cognitive, motor, and social stimulation [20]. The essential components of an EE, such as moderate aerobic exercises, can help decrease glycemic oscillation in patients with type 2 diabetes [21]. Even short-term exercises (less than two weeks) decrease the mean amplitude of glycemic excursion in patients with type 2 diabetes [22], and it is known that glucose variability is an important worsening factor of DN [23]. Recent studies report that, while basal neurotransmission remained unaffected by EE, synaptic plasticity and long-term potentiation (LTP) were significantly affected [24]. Glutamate is primarily involved in LTP via reactive oxygen species (ROS) generation [25]. Oxidative glutamate toxicity or oxytosis (oxidative stress-dependent non-apoptotic cell death) is an important source of oxidative stress and the depletion of glutathione [26] and a crucial pathological mechanism of neuronal disorders [27]. Although generally broadly discussed mainly for type 2 diabetes, data also exist about glutamine metabolism disturbances in type 1 diabetes [27,28]. Interestingly, some glutamatergic neurons have been found to express GLP-1 receptors, and liraglutide modulates its activities [29].

Studies of the potential of the early treatment of DN can inform us about early mechanisms with translational potential. So, we focused on the early effects of a promising antidiabetic drug, liraglutide, and its potential analgesic, anti-inflammatory, and antioxidative stress effects. Neopterin is a product of interferon-gamma (IFN-γ) activation of macrophages and has been proposed as a serum marker for peripheral neuropathy in diabetes mellitus [30]. It has been shown that pteridine, a precursor of neopterin, can be produced by nerve cells under oxidative stress [31]. Macrophages also possess GLP-1 receptors [30], indicating an interesting potential target for liraglutide. This study aimed to evaluate the potential of the agonist of GLP-1 receptors, liraglutide, in the early management of DN in two mouse models, i.e., under standard conditions [32] and in an EE. The study also explored the potential implications of glutamine/glutamate and neopterin in this context, informing about the potential interplay of the incretin system with oxidative stress, assessed by the glutamine-to-glutamate ratio and the inflammatory marker neopterin.

## 2. Results

### 2.1. Body Weight Gain/Loss during the Trial

For the 10-day study, mice from the nondiabetic control group (C) gained 3.03% of their BW, whereas those from the three diabetic groups, namely D (diabetic controls), DLS (diabetics, liraglutide-treated, under standard conditions), and DLE (diabetics, liraglutide-treated, under EE), lost 14.30%, 9.38%, and 16.12%, respectively—Table 1. When testing for the factor “Group”, significant differences were revealed: H(3) = 21.61, *p* < 0.001, ƞ^2^ = 0.39 (large effect). All diabetic mice lost some body weight (BW), irrespective of the 10-day liraglutide treatment.

### 2.2. Antinociceptive Behavior

#### 2.2.1. Formalin Test 

Typically, after the formalin injection, the mouse start intensively licking or biting the injected place. A quiet period follows, and after approximately 20 min the behavior reoccurs.

The analysis of the first five minutes post-formalin injection (phase 1) showed statistically significant differences between groups (F (3, 44) = 11.271 *p* < 0.001 ƞ^2^ = 0.28 (large effect)). Intergroup analysis revealed that this difference was due to group D’s differing from groups C (*p* = 0.047), DLS (*p* = 0.002), and DLE (*p* < 0.001), suggesting that mice with untreated diabetes experienced higher sensitivity to the chemical stimulus. On the other hand, liraglutide treatment alleviated the sensation of pain, as seen by decreased analgesic behavior.

**Figure 1:**
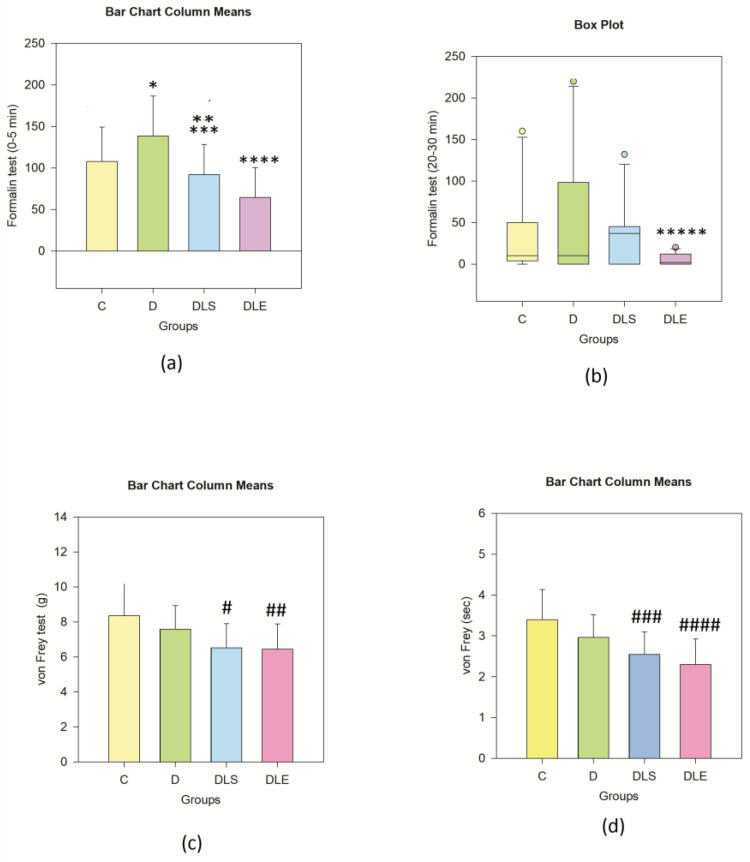
Antinociceptive behavior: (**a**) Phase 1,0 seconds after formalin hind paw s.c. injection; (**b**) Phase 2 of the formalin test; (**c**) von Frey test—pressure force; (**d**) von Frey test—speed of reaction (seconds). n = 12 in each group. Significantly longer antinociceptive behavior during the first 5 min of the test was recorded in group D, as compared to all other groups. * *p* = 0.047 for D vs. C; ** *p* = 0.002 for D vs. DLS; *** *p* = 0.024 for DLE vs. C. In the group for DLE vs. D, **** *p* < 0.001. Results for the 1st phase were obtained with one-way ANOVA with Tukey post hoc due to the normality of the distribution of those values. Significantly lower antinociceptive behavior was recorded in the second phase of the formalin test for DLE vs. D (***** *p* < 0.05) when tested with Kruskal–Wallis and Dunn’s post hoc, due to the lack of the Gaussian distribution of those values. The von Frey test demonstrated the enhanced reactivity of the two liraglutide-treated diabetic groups, irrespective of the rearing conditions (# *p* = 0.023 for C vs. DLS; ## *p* = 0.002 for C vs. DLE), when measured in grams of the applied pressure or in seconds from stimulus-to-reaction (### *p* = 0.009 for C vs. DLS; #### *p* < 0.001 for C vs. DLE). The von Fray test was assessed using one-way ANOVA and a Tukey post hoc test.

Mice reared in the EE demonstrated significantly lower antinociceptive behavior than controls (*p* < 0.024). These results suggest a beneficial effect of liraglutide treatment on diabetic animals—Figure 1a, Table 2. No statistical difference was noticed between the DLS and DLE groups.

From the measurement of the antinociceptive behavior between the 20th and the 30th min of the formalin test (phase 2) (Figure 1b, Table 2), again, groups differed in a statistically significant manner (H(3) = 11.983, *p* = 0.007, ƞ^2^ = 0.20 (large effect)), and this was at the expense of group D (non-treated diabetic mice) as opposed to DLE (liraglutide-treated, reared in EE, with diabetes) (*p* < 0.05). The decreased antinociceptive reaction during the second phase of the formalin test indicates that mice had lower sensitization under the concomitant effect of the liraglutide treatment and EE.

#### 2.2.2. Von Frey Test

Animals were tested for allodynia by applying non-painful stimuli with a dynamic plantar esthesiometer for the automatic von Frey test (Ugo Basile, Italy). Regarding the pressure at which the animal responded, the results showed that the groups differed statistically significantly—F (3, 44) = 6.079, *p* = 0.011 ƞ^2^ = 0.17 (large effect)—Figure 1c, Table 2. Here, we are reporting an increased sensitivity toward non-painful stimuli of mice from the liraglutide-treated groups DLS vs. C (# *p* = 0.023) and DLE vs. C (## *p* = 0.002).

A study of the reaction time during the von Frey test demonstrated similar results, namely statistically significant differences among groups with F (3, 44) = 7.289, *p* < 0.001, ƞ^2^ = 0.26 (large effect) and a statistically significant increased reactivity of the two liraglutide-treated groups DLS (*p* = 0.009) and DLE (*p* < 0.001) vs. controls (C). The two liraglutide-treated groups had comparable increased reactivity in the von Fray test.

### 2.3. Biochemistry

#### 2.3.1. Blood Glucose

**Figure 2:**
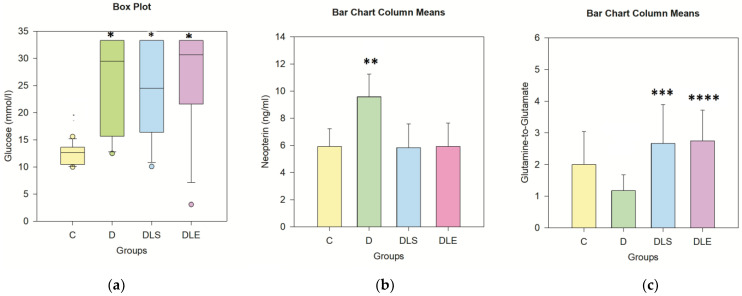
(**a**) Fasting blood glucose at the end of the trial, (**b**) serum neopterin, (**c**) serum glutamine-to-glutamate ratio. n = 12 for each group. All groups with diabetes, i.e., D, DLS, and DLE, were hyperglycemic (* *p* < 0.05). Neopterin level was significantly higher in the group with untreated diabetes (group D) vs. all other groups (** *p* < 0.001). The glutamine-to-glutamate ratio was significantly increased in the two groups treated with liraglutide: *** *p* = 0.003 for DLS and **** *p* = 0.002 for DLE versus D. Data were tested with Kruskal–Wallis, Dunn’s post hoc (for glucose) or one-way ANOVA, and Tukey post hoc (for neopterin and glutamine-to-glutamate) tests.

Data from the blood glucose testing were non-normally distributed, so Kruskal–Wallis ANOVA was applied—Figure 2a, Table 2. Differences in median values between groups were statistically significant H(3) = 18.54, *p* ≤ 0.001, ƞ^2^ = 0.30 (large effect). As per Dunn’s test, all streptozotocin-treated mice had blood glucose values significantly higher than controls (*p* < 0.05) irrespective of the liraglutide treatment under the standard environment or the EE.

#### 2.3.2. Serum Neopterin

Neopterin levels significantly differed between groups: F (3, 47) = 15.48, *p* < 0.001, 2 = 0.30 (large effect)—Table 3, Figure 2b. The difference was due to the significantly higher neopterin level in the diabetic control group (D) than in the control group C (*p* < 0.001). Liraglutide treatment mitigates this effect in both DLS and DLE groups, and significantly lower neopterin levels were recorded in DLS and DLE (*p* < 0.001).

#### 2.3.3. Glutamine/Glutamate

For the glutamine-to-glutamate ratio (Table 3, Figure 2c), a statistically significant difference among groups was also revealed: F (3, 47) = 6.76, *p* < 0.001, ƞ^2^ = 0.19 (a large effect). Both liraglutide-treated groups significantly differed from the diabetic control group, suggesting that the increased glutamine-to-glutamate ratio is an important liraglutide effect (*p* = 0.002 for DLE vs. D and (*p* = 0.003 for DLS vs. D). Again, no differences were found as a result of rearing conditions.

## 3. Discussion

### 3.1. Short-Term Effect of Liraglutide in Streptozotocin-Induced Type 1 Diabetes 

In this study, ten days after the streptozotocin injection, statistically significant hyperglycemia and weight loss, polydipsia, and polyuria were observed in all streptozotocin-injected mice. In our case, the rate of acute streptozotocin toxicity, which ordinarily occurs up to 1 week post-injection, was 20%, was equally so for the three streptozotocin groups.

Two treatment groups were used to assess the liraglutide therapeutic potential in standard conditions or the EE. As expected, in our study, liraglutide did not improve glycemia either in the DLS or in the DLE group. It also did not change the lower BW of mice with diabetes, regardless of the rearing conditions. As was demonstrated by other authors, liraglutide has no effect on body weight, food intake, or body fat in non-obese mice [33]. The incretin mimetic liraglutide supposedly goes to the pancreas to increase prandial insulin and suppress glucagon secretion [34]. Streptozotocin, however, destroys beta cells in the pancreas [35], compromising liraglutide’s insulin secretagogue activity. Earlier studies reported that streptozotocin-induced beta-cell toxicity can be observed as early as within 72 h [36].

### 3.2. Short-Term Effect of Liraglutide in Antinociceptive Behavior in Streptozotocin-Induced Diabetic Neuropathy

Irrespective of the ineffectiveness of liraglutide in mitigating the blood glucose level in streptozotocin-treated mice, we demonstrate a decrease in the antinociceptive behavior in the formalin test, suggesting the amelioration of DN. With this study, a diabetic model of neuropathy was assessed both with the formalin test and the von Frey test in mice bred under a standard or EE. Reportedly, animals housed in an EE do not show more significant variability than those reared under standard conditions. Therefore, environmental heterogeneity introduced in the laboratory, in the form of enrichment, does not compromise the integrity of the data but, on the contrary, increases its credibility [37].

Other investigators demonstrated the impairment of sensory conduction velocity in the tail nerve as early as seven days post-streptozotocin injection [38]. However, the translatability of this finding to humans needs further clarification due to the lack of a respective organ in humans. In our trial, however, the DN demonstrated antinociceptive behavior during the formalin test 10 days after the diabetes induction.

### 3.3. Short-Term Liraglutide Treatment Demonstrates Different Behavior Antinociceptive Effects in the Formalin Test in Standard or EE-Reared Mice

In our trial, the beneficial effect of the EE in combination with liraglutide significantly decreased the second phase of the formalin test (D vs. DLE *p* < 0.05), not seen for D vs. DLS. The two phases of the formalin test provide different information about putative underlying mechanisms. The first phase demonstrates the behavioral reaction toward an acute excitation of nociceptors, whereas the second phase indicates central sensitization. Compared with the von Fray test, the formalin test better differentiated liraglutide-treated groups from the D group in both test phases. Different mechanisms, for example, drive the early and late phases of the formalin test, transient receptor potential-1 (TRP1) ion channel activation (for about the first 5 min of the test), and inflammation with central sensitization [39] or excitotoxicity of the skin [40] (the late second phase of the test). Our study confirms the beneficial effects of liraglutide in streptozotocin-induced neuropathy in mice, assessed as a decrease in antinociceptive behavior.

Conventional laboratory cages for rodents can lead to the development of stereotyped behavior, weakened immunity, increased acute hormonal reactions to stressors, and behavior indicating anxiety and pain [41]. The beneficial effects of EE rearing are reported in a rodent model of paclitaxel-induced neuropathy [42], with chronic damage to the sciatic nerve [43], with spinal cord injury [44], both in terms of pain and depressive-like behavior and memory [45].

### 3.4. Short-Term Liraglutide Treatment Effects on Neopterin and Glutamine-to-Glutamate Ratio

Aiming to explore further the pathogenic mechanisms influenced by liraglutide’s effect, we evaluated serum glutamine/glutamate and neopterin as factors directly or indirectly characterizing inflammation and oxidative stress. Diabetes is a metabolic disorder associated with chronic inflammation and oxidative stress. Unsurprisingly, all investigations focus on these two phenomena when approaching deeper mechanisms of action of liraglutide.

In vitro experiments demonstrate that liraglutide reduces oxidative stress and ameliorates energy metabolism [46], attenuates hepatic oxidative stress and inflammation [47], and increases glutathione concentration in the serum of patients with type 2 diabetes [48]. Here, we report that mice treated with liraglutide present significantly higher serum glutamine-to-glutamate vs. the D group, irrespective of rearing under standard conditions or an EE. Data from a recent comparative metabolome analysis of mice demonstrate that liraglutide decreases the pentose phosphate pathway but elevates the urea cycle and ammonia recycling [49]. Glutamate and glutamine play an important role in ammonia recycling. Glutamine is the most abundant free amino acid in the body, and it plays a role in multiple pathways that impact blood glucose in either direction. Glutamine-to-glucose gluconeogenesis accounts for up to 8–16% of the glucose in a fasting state, glutamine being essential for intestinal gluconeogenesis [50], a process found to be enhanced in animal models of diabetes [51]. The particularity of glucose produced by the intestine (compared with the liver and kidney) is that it is detected in the portal vein and initiates a nervous signal to the hypothalamic nuclei regulating energy homeostasis [52]. Our experiment further confirmed that a diabetic state is related to lower glutamine/glutamate levels.

Glutamine is a precursor of glutathione, the most abundant intracellular antioxidant. Glutathione substantially decreases both in type 1 diabetes and type 2 diabetes [53]. In streptozotocin-treated rats, L-glutamine supplementation attenuated the development of experimental diabetic cardiomyopathy [54]. Glutamine is a precursor of citrulline, which is converted to arginine in the kidneys by the enzyme argininosuccinate synthetase [55]. Arginine is the sole precursor of nitric oxide in the body, and its serum levels are higher in patients with diabetes than in those with controls. In low concentrations, nitric oxide mediates the insulin secretagogue action of L-arginine, whereas high nitric oxide exerts the opposite effect and destroys beta cells [56]. Glutamine also promotes the secretion of the incretin glucagon-like peptide-1 (GLP-1) in healthy subjects as well as in those with type 2 diabetes [57], and activated glutamate dehydrogenase is an important governor of this [58].

Glutamate dehydrogenase is essential to sustain neuronal oxidative energy metabolism during stimulation [59]. In the neuronal tissue, glutamine is a precursor of two neuro mediators, glutamate and γ-aminobutyric acid (GABA) [60]. GABA may produce neuroendocrine, autonomic nervous system, and metabolic counterregulatory failure during exercise-induced hypoglycemia in patients with type 1 diabetes [61]. On the other hand, glutamate, as a main excitatory neurotransmitter, via the overstimulation of its ionotropic NMDA receptors, may increase Ca^2+^ influx into neurons together with the generation of oxidative/nitrosative stress and finally damage the cell [62]. Other investigators reported that NOX, but not mitochondrion, is neurons’ primary source of NMDA-induced superoxide production [63].

Human neurons and astrocytes secrete neopterin under inflammatory conditions [64]. Recently, analgesic, anti-inflammatory, and anti-degradative actions of liraglutide in mouse models of osteoarthritis were reported [65]. Here, we demonstrated that neopterin levels of non-treated liraglutide diabetic mice are statistically higher, and the glutamine/glutamate ratio is significantly lower compared to liraglutide-treated mice. On the other hand, treatment with liraglutide was associated with lower neopterin and higher glutamine-to-glutamate levels. No difference was observed between mice bred in a standard vs. EE.

Data about the diagnostic values of serum neopterin in patients with diabetes differ. In general, patients with diabetes have higher serum neopterin than healthy subjects [66]. For those with type 1 diabetes, neopterin seems to be a valuable early biomarker for DN [30]. However, recent studies fail to confirm this for type 1 diabetes [67] and type 2 diabetes [68]. In patients with diabetic foot syndrome, however, neopterin levels are higher in patients with type 2 vs. type 1 diabetes [69]. Here, we created a model of type 1diabetes and demonstrated significantly increased neopterin in the D group, differing from the liraglutide-treated mice. Neopterin is a broad-ranging inflammatory marker, but it is also a key antioxidant released by the immune cells [70]. Reportedly, neopterin suppresses the main culprit for oxidative stress in neurological disorders, the membrane-bound NADPH oxidase (NOX) [71]. We could not find a published study of the effects of liraglutide on the neopterin level in streptozotocin neuropathy models in rodents.

### 3.5. Limitations

Our study has several limitations. Only male ICR mice were evaluated. The study had a short-term duration. Longer experiments will provide more detailed information about liraglutide’s therapeutic potential in DN. Although we admit that, from the point of view of the assessment of neopterin and glutamine/glutamate levels, groups of nondiabetic and diabetic mice would be of interest, our study focused mainly on liraglutide’s effects, so we decided not to include such a separate group. This study reports the effect of liraglutide on the serum glutamine-to-glutamate ratio and neopterin together with behavior antinociceptive effects. Although we suppose that DN in our study is mostly related to functional rather than morphological neuronal impairment (an early stage of neuropathy), a better characterization of the effect of the liraglutide on the peripheral nerves could be obtained with additional histopathology testing.

## 4. Materials and Methods

### 4.1. Chemicals and Kits

Streptozotocin (S0130), liraglutide (SML3925), ketamine hydrochloride/xylazine hydrochloride solution (K4138), formalin solution, neutral buffered, 10% (HT5011) and a Glutamine and Glutamate Determination Kit (J80210) were acquired from Sigma-Aldrich, St. Louis, MO, USA. Citrate buffer pH 4.5 was from Acros Organics, Geel, Belgium. A Mouse Neopterin ELISA kit MBS2600658-96 was obtained from MyBioSource, San Diego, CA, USA.

### 4.2. Experimental Design

Male ICR mice, 20–25 g (6–7 weeks of age), were received from the vivarium of the Medical University, Sofia. They were randomly distributed to five mice in a cage by an animal caregiver who was unrelated to the experiment and unfamiliar with the treatment of the groups. A few days after the establishment of their social hierarchy, 45 mice were subjected to overnight fasting and injected intraperitoneally with streptozotocin at a single dose of 150 mg/kg freshly dissolved in citrate buffer (pH 4.5) as per a published protocol to create a type 1diabetes state [72]. After returning the mice to their cages, the food and 10% glucose solution were provided ad libitum. Three days thereafter, the glucose solution was replaced with fresh water.

**Figure 3:**
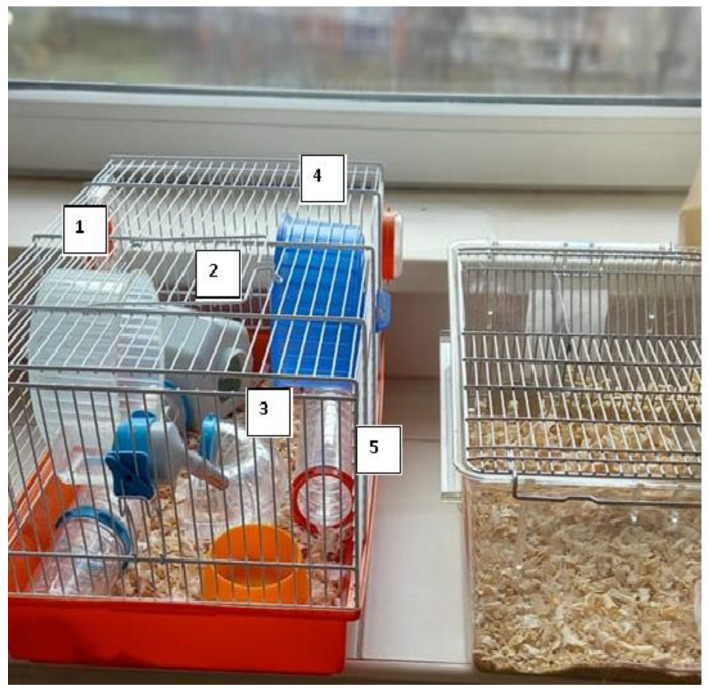
**A** visual representation of the environmental conditions used for mouse rearing. The enriched environment (**left**) included elements such as rotating wheels (1, 4), an igloo-type house (2), and tunnels (3, 5), compared to the standard housing without additional stimuli (**right**). This setup was used to differentiate between standard and enriched living conditions for mice.

From streptozotocin-injected mice, 30 were placed in two different cages: standard laboratory cages (30 × 17 × 16 cm) with only basic bedding materials and larger cages (60 × 60 × 30 cm) to create two types of rearing conditions (standard and EE). The EE was designed to stimulate physical and cognitive activities, providing larger cages equipped with tunnels, igloo-style houses, and rotating wheels. These objects were rearranged every other day to maintain novelty and encourage exploration—Figure 3.

Standard laboratory chow and water ad libitum were supplied. A total of five mice were housed per cage, both in standard and EE conditions, with the ambient temperature set at 22 °C and light–dark cycles maintained for 12 h each. To minimize the suffering of the animals, they were regularly monitored for signs of distress or discomfort. Daily inspections assessed murine grooming, physical appearance, and social interaction. In addition, a veterinarian was on call to assess any signs of abnormal behavior or severe weight loss, and humane euthanasia was performed when necessary, according to institutional ethical guidelines. The mortality following streptozotocin injection was 20%, similar in mice bred in the standard and EE. The development of diabetes was accompanied by significant polydipsia, polyuria, polyphagia, and significant weight loss.

On the second week of the streptozotocin injection, mice were randomly grouped by an animal caregiver, nonrelated to the experiment, as follows:

- C—control group raised under standard conditions in standard Plexiglas cages (n = 12).
- D—a group with developed diabetes, grown in standard conditions in standard Plexiglas cages (n = 12).
- DLS—a group with developed diabetes, bred in standard Plexiglas cages and treated with liraglutide (n = 12).
- DLE—a group with developed diabetes, bred in an EE, treated with liraglutide (n = 12).

In the following ten days, the DLS and DLE groups started to receive a once-daily injection of liraglutide (0.4 mg/kg, in saline) intraperitoneally in a volume of 0.1 mL/10 g BW, and groups C and D were injected with saline only. The dose was selected as per the previous experiment, using 400 μg/kg in type 1 diabetes mice. The treatment duration was based on the reported effective neuroprotective dose for a period as short as seven days [73,74]. Reportedly, an EE also favorably affects cognitive deficits in the streptozotocin model of diabetes mellitus [75], and short-term exposure to the EE, for about ten days, seems sufficient [76]. To decrease potential confounding factors, we ensured mice were handled by the same five experienced investigators in the same room.

### 4.3. Body Weight Gain/Loss during the Trial

Mice were weighed twice, i.e., at the start (before the streptozotocin injection) and at the end of the trial (after ten days of liraglutide application). The weight gain/loss at the end of the trial was calculated for each mouse by subtracting the weight (g) at the end of the trial from the initial weight (g) and expressed as a % from the basal value.

### 4.4. Behavior Tests

After 10 days of liraglutide treatment, in the morning (from 8.30 to 11.30), the behavior test were performed. All animals were naïve to each pain test.

#### 4.4.1. Formalin Test

A mouse formalin test of hyperalgesia, as per the publicly available protocol from the website of the National Institute of Neurological Disorders and Stroke, was applied (https://panache.ninds.nih.gov/Home/LegacyModels, accessed on 10 May 2024). Formalin (0.5%) was injected into the right hind paws of the mice, and the animals were placed in individual Plexiglas observation cages. In this test, we recorded two phases of the animals’ antinociceptive behavior (licking, biting, taking an antalgic posture): an early phase, during the first 5 min, and a late phase, from the 20th to the 30th minute after the formalin injection. Two independent observers counted the time spent on analgesic reactions.

#### 4.4.2. Von Frey Test (Dynamic Plantar Aesthesiometer)

Mechanical sensitivity was evaluated using the Dynamic Plantar Aesthesiometer (Ugo Basile, Italy), as per manufacturer instructions (https://nbtltd.com/wp-content/uploads/2018/10/37450_r1.pdf). The apparatus applies a standardized filament force ranging from 0.1 to 100 g perpendicularly to the plantar surface of the paw, and the force increases gradually by 0.1 g increments until a withdrawal response is automatically detected. The system ensures consistency in force application without the need for manual adjustments, thereby eliminating the need for the traditional “up-and-down method”.

The left hind paw of the mice was tested to exclude interference with the formalin test. Animals were placed one at a time on the small raised platform with small mesh bottom cells of the apparatus, and after habituating for 30 min, they were touched by the automatic monofilament (0.5 mm) perpendicular to the plantar surface of the hind paw using the ‘ascending stimulus’ method. In this stimulus, monofilaments of increasing force were applied until a withdrawal response was elicited, and the monofilament force that elicited a positive response (a sharp withdrawal of the paw) was recorded as the mechanical withdrawal threshold. Each animal underwent three trials, and the average mechanical withdrawal threshold (in grams) and latency to paw withdrawal (in seconds) were calculated automatically. The automated nature of the test minimized experimenter bias and provided highly reproducible and sensitive results.

### 4.5. Biochemistry

#### 4.5.1. Pre-Analytical Stage

The day following the behavioral tests, at 6.00 a.m., the food was removed from the cages. Six hours later, animals were anesthetized with ketamine (100 mg/kg i.p.) and xylazine (10 mg/kg i.p.), and blood was collected by cardiac puncture. Euthanasia was performed with exsanguination.

A drop of whole blood was used for glucose testing, and the remaining blood was collected in vacutainers with a gel-separator, centrifugated at 1800× *g* for 10 min, and the serum was frozen at −20 °C until the evaluation.

#### 4.5.2. Blood Glucose Testing

The glucose level was determined immediately from the heart puncture during the euthanasia procedure. Fasting whole blood glucose levels were tested with strips and a Wellion Luna trio glucometer (Wellion, Austria).

#### 4.5.3. Neopterin Evaluation

The determination of neopterin in serum was performed with the Mouse Neopterin ELISA kit MBS2600658-96 (MyBioSource, USA) according to the manufacturer’s instructions, using the double-antibody-sandwich ELISA method. The assay uses an anti-mouse neopterin monoclonal antibody and a biotinylated poly antibody for detection after adding 100 mcl of the samples. After washing with phosphate buffer, tetramethylbenzadine (TMB) substrate is added, which reacts with avidin-peroxidase to produce a color reaction (blue product), which changes the color of the samples to yellow when the stop solution is added. The reading is spectrophotometric at 450 nm at the 10th minute.

#### 4.5.4. Glutamine/Glutamate Evaluation

Serum glutamine–glutamate determination was performed using the Glutamine and Glutamate Determination Kit (J8021-Sigma-Aldrich, USA) according to the manufacturer’s instructions. This kit spectrophotometrically measures L-glutamine and/or L-glutamate by the enzymatic deamination of L-glutamine and the dehydrogenation of L-glutamate with the conversion of NAD+ to NADH. Determining L-glutamine is a two-step reaction, featuring (1) the deamination of L-glutamine to L-glutamate and (2) the dehydrogenation of L-glutamate to α-ketoglutarate, accompanied by reducing NAD+ to NADH. The conversion of NAD+ to NADH is measured spectrophotometrically and is proportional to the amount of oxidized glutamate; hence, this explains the amount of glutamine converted to glutamate in the samples. Since both glutamine and glutamate are expected to be present in the serum, endogenous glutamate should be measured. In this case, the first step is the determination of glutamine by a glutamine deamination reaction. As a result of this step, the total content of glutamine and glutamate in the sample is reported. From this, the value obtained in the dehydrogenase reaction from the amount of glutamate in the sample was subtracted. The reading was at 340 nm after 40 min.

### 4.6. Statistics

Data are presented as the mean ± standard deviation, when Gaussian distributed, or as the median and interquartile ranges. Statistical analysis was performed using SigmaPlot v.15 software. This software automatically performs pre-tests for the normality of the distribution and the equality of variances, which are the important preconditions to apply the parametric ANOVA. Otherwise, non-parametric Kruskal–ANOVA was applied. In the case of statistical significance, Tukey’s or Dunn’s post hoc test were applied. Values of *p* < 0.05 were considered statistically significant.

Two-way repeated measure ANOVA was used to analyze the BW at the end vs. at the start of the experiment, followed by Dunn’s post hoc test.

The effect size was calculated as (partial) eta squared from SSeffect/(SSeffect +SSerror) or as (H-k + 1)/(n-k) for non-parametric ANOVA. A small (up to 0.01), medium (from 0.06), or large (from 0.14) effect was used as classification.

## 5. Conclusions

Despite our knowledge of multiple mechanisms, our options for managing DN remain scarce. This study demonstrates the beneficial effect of liraglutide in two streptozotocin-induced mouse diabetes models. Despite substantially high blood glucose levels, we suggest that liraglutide improves neuropathic behavior in streptozotocin-treated mice, interfering with inflammation and oxidative stress. Our findings can be translated to human treatment. Liraglutide is an approved drug for diabetes and obesity treatment, with known beneficial effects on the cardiovascular complications of diabetes. We can expect that at least two different targets, hyperglycemia, and the microcirculation of peripheral nerves, can ameliorate the DN in humans. Future research, including longer treatment durations, different dosages, the inclusion of female mice, and the exploration of another potential therapeutic target, would be beneficial to improve the translatability of recent findings to DN management in humans.

## Figures and Tables

**Table 1:** Body weight by treatment groups at the start and end of the trial.

Groups	Time	Body Weight (g)	Weight Gain/Loss (g)	% from the Initial BW Value for Each Mouse
C	Start	33.00 (30.25 ÷ 34.00)		
	End	33.00 (31.00 ÷ 34.00)	1.00 (0.00 ÷ 2.00)	+3.03 (−2.14 ÷ 5.27)
D	Start	33.50 (30.50 ÷ 35.50)		
	End	28.00 * (26.00 ÷ 30.00)	−5.50 * (−8.50 ÷ −3.50)	−14.30 * (−21.71 ÷ −10.34)
DLS	Start	33.00 (30.25 ÷ −35.00)		
	End	29.00 (25.00 ÷ 33.50)	−3.50 * (−7.00 ÷ 0.00)	−9.38 * (−27.19 ÷ +3.00)
DLE	Start	34.00 (31.00 ÷ 35.50)		
	End	30.00 (25.00 ÷ 32.00)	**−3.50** * (−5.00 ÷ −2.50)	−16.12 * (−19.30 ÷ −9.38)

Statistical significance was *p* < 0.05 for comparisons between the D group at the end versus the start of the trial, as well as for comparisons between the D, DLS, and DLE groups versus the control group (C). A Kruskal–Wallis test followed by a Dunn’s post hoc test was used for analysis, with n = 12 mice in each group. Data are shown as medians with interquartile ranges. The bold indicates statistically significant values. * indicates p < 0.05.

**Table 2:** Antinociceptive behavior.

Groups	Formalin Test (s)		Von Frey Test	q
	0–5 min	20–30 min	(grams)	(s)
C (n = 12)	108.25 ± 37.79	10.00 (8.00–13.00)	8.48 ± 1.90	3.40 ± 0.74
D (n = 12)	147.92 * ± 35.64	33.00 (10.00–124.00)	7.59 ± 1.35	3.00 ± 0.55
DLS (n = 12)	91.83 ** *** ± 34.70	29.00 (2.25–41.50)	6.58 # ± 1.36	2.55 ### ± 0.55
DLE (n = 12)	64.50 ***** ± 35.80	2.00 ***** (0.00–11.00)	5.96 ## ± 1.58	2.30 #### ± 0.62

* *p* = 0. The significantly prolonged 1st phase of the formalin test in D group (* *p* = 0.047) vs. controls (C). The significantly decreased 1st phase in both liraglutide-treated groups, DLS and DLE (** *p* = 0.002 and *** *p* = 0.024) vs. controls. The significantly lower antinociceptive behavior in the DLE vs. D group (***** *p* < 0.05) of the 2nd phase of the formalin test. The von Frey test demonstrates increased sensitivity towards non-painful stimuli of mice from the DLS (# *p* = 0.023) and DLE (## *p* = 0.002) groups, expressed in grams, or seconds (### *p* = 0.09 or #### *p* < 0.001), for DLS or DLE vs. C respectively).

**Table 3:** Biochemical parameters.

Groups	Glucose (mmol/L)	Neopterin (ng/mL)	Glutamine/Glutamate
C (n = 12)	12.65 * (10.55–13.55)	5.92 ± 1.31	2.00 ± 1.04
D (n = 12)	29.5 * (16.65–33.30)	9.58 ** ± 1.68	1.17 ± 0.50
DLS (n = 12)	24.5 * (16.60–33.30)	5.83 ± 1.75	2.67 *** ± 1.23
DLE (n = 12)	30.65 * (23.05–33.30)	5.92 ± 1.73	2.75 **** ± 0.96

For blood glucose testing, * *p* < 0.05 for comparisons between D (control group with diabetes), DLS (a group with diabetes, treated with liraglutide in a standard environment), and DLE (liraglutide-treated mice with diabetes reared in the EE) versus the control group (C). Statistical analysis was performed using Kruskal–Wallis ANOVA, followed by Dunn’s post hoc test. Glucose data are presented as medians with interquartile ranges, For neopterin testing, ** *p* < 0.001 for D versus C, DLS, and DLE groups. For glutamine-to-glutamate testing, *** *p* = 0.003 was revealed for D versus DLS; **** *p* = 0.002 for D versus DLE. One-way ANOVA with Tukey’s post hoc test was used for neopterin and glutamine/glutamate data, which are shown as means with standard deviations. The bold indicates statistically significant values.

## Data Availability

The original contributions presented in the study are included in the article; further inquiries can be directed to the corresponding author.
